# A highly secure method for rearing *Aedes aegypti* mosquitoes

**DOI:** 10.1186/s41182-018-0098-5

**Published:** 2018-05-23

**Authors:** Manabu Ote, Hirotaka Kanuka

**Affiliations:** 1 Department of Tropical Medicine, The Jikei University School of Medicine, Tokyo, Japan; 2 Center for Medical Entomology, The Jikei University School of Medicine, Tokyo, Japan

**Keywords:** Mosquito, Vector, Pathogen, Arbovirus, Blood-sucking, Hematophagy, Security

## Abstract

**Background:**

Vector-borne infectious diseases are caused by pathogenic microorganisms transmitted mainly by blood-sucking arthropod vectors. In laboratories, the handling of insects carrying human pathogens requires extra caution because of safety concerns over their escape risk. Based on standard insect containment practices, there have been cases where costly enhancements were required to definitely protect laboratory workers and neighbors from potential infection through mosquito bites. Here, we developed a mosquito rearing method that provides a reliable and cost-effective means to securely contain pathogen-infected females of the yellow fever mosquito *Aedes aegypti*.

**Results:**

To debilitate the motility of *A. aegypti* females, mosquitoes were rendered completely flightless by ablation of either wing. The “single-winged” mosquitoes exhibited a severe defect in flying ability and were incubated in a container with inside surfaces covered with a net stretched to approximately 1-mm mesh, which helped the mosquitoes hold on and climb up the wall. In this container, flightless females consistently showed similar blood feeding and egg laying activities to intact females. Eighty-five percent of the flightless mosquitoes survived at 1 week after wing ablation, ensuring feasibility of the use of these mosquitoes for studying pathogen dynamics.

**Conclusions:**

This mosquito rearing method, with a detailed protocol, is presented here and can be readily implemented as a highly secure insectary for vectors carrying human pathogens. For researchers in an environment where highly strict containment practices are mandatory, this method could offer appropriate opportunities to perform research on pathogen–mosquito interactions in vivo.

**Electronic supplementary material:**

The online version of this article (10.1186/s41182-018-0098-5) contains supplementary material, which is available to authorized users.

## Background

Vector-borne infectious diseases such as dengue fever and malaria account for one sixth of all illnesses and disabilities around the world, leaving more than half the world’s population at risk [1]. Disease-causing microorganisms, virus, bacteria, protozoan parasites, and worms are transmitted from host animals to humans mainly through blood-sucking arthropod vectors. The vectors can perceive multiple sensory cues including emitted odor, carbon dioxide, and body heat to precisely locate their hosts and feed on blood. In particular, highly motile flying insects such as mosquitoes and flies have the ability to disperse over a long range and efficiently transmit pathogens.

Microorganisms originating from host blood are amplified and disseminated inside vectors, thereafter migrating into the mouthparts, settling in the throat, or invading salivary glands for transmission into new hosts. One of the important measures possibly leading to the success of blocking pathogen transmissions is to understand the vector–pathogen interactions underpinning the pathogen burden in vectors. One of the major impediments for research on pathogen–vector interaction is derived from safety concerns regarding the risk of the inadvertent escape of vectors carrying human pathogens when infectious agents are introduced into vectors. In the case of being on the loose, the vectorial capacity of flying insects associated with high motility and excellent host perception could be a serious threat for laboratory workers. Infected workers could unintentionally become a potential source of pathogens in areas where adequate vectors capable of transmission are indigenous in the wild.

The World Health Organization and others have published laboratory biosafety manuals, which provide practical guidance on biosafety techniques [2, 3]. The insectary should be designed in accordance with the biosafety level determined by the risk group of the pathogen. In addition, appropriate precautions are necessary to manage flying vectors (mostly mosquito species) infected with human pathogens, including containment conditions in which the infectious agents should be safely handled (e.g., multiple doors and air shower booth). Moreover, in keeping with institutional guidelines, risk assessments for the safety of laboratory workers and neighbors should be implemented during infection experiments. In any case, comprehensive safety measures to secure any people should be prepared regardless of the technical proficiency of the workers. It is, therefore, necessary to develop additional equipment and/or methods for handling pathogen-infected flying vectors more securely.

Here, we describe an improved method for rearing mosquitoes without the risk of escape, which thereby robustly secures the safety of people in laboratory. Female mosquitoes are rendered flightless by cutting off either wing, resulting in little adverse effect on their fitness. The attenuated mobility of the flightless females makes it less laborious to implement strict containment processes. This procedure proposed here can mitigate the risk of inadvertent infection of laboratory workers and neighbors when applied to research involving mosquitoes carrying human pathogens.

## Methods

### Ethics statement

This study was carried out in accordance with the Guideline for Laboratory Animals of The Jikei University School of Medicine and the Fundamental Guidelines for Proper Conduct of Animal Experiment and Related Activities in Academic Research Institutions of the jurisdiction of the Japanese Ministry of Education, Culture, Sports, Science, and Technology. The protocol was approved by the Committee on the Animal Experiments of The Jikei University School of Medicine (Permit Number 2016-051).

### Rearing of *A. aegypti* mosquitoes

The *A. aegypti* strain employed in this study originated from the Liverpool strain and was a gift from Dr. Ryuichiro Maeda (Obihiro University of Agriculture and Veterinary Medicine). Eggs obtained using a standard procedure were hatched in reverse osmosis (RO) water. The first instar larvae were transferred to a plastic container with RO water and daily fed with fish food (Kyorin Co., Ltd., Hikari [#4971618-013378]). The larvae were kept in an insectary room set at 27 °C, and water in the container was refreshed every 2–3 days. Pupae were harvested in a plastic cup and placed within a cage (bottom 27 cm × 27 cm, top 25 cm × 25 cm, height 27 cm), in which a filter paper (Whatman, Grade 4, 1004-125) soaked in 10% sucrose solution was placed in a 50-mL glass flask. The cage was kept in an incubator (Panasonic, MIR254-PJ) set at 27 °C with humidity over 90% in cycles of 12 h light and 12 h darkness. The cup left in the cage was put out 2 days after starting incubation. The sucrose solution was changed every 3–4 days.

### Preparation and maintenance of flightless mosquitoes

**Fig. 1:**
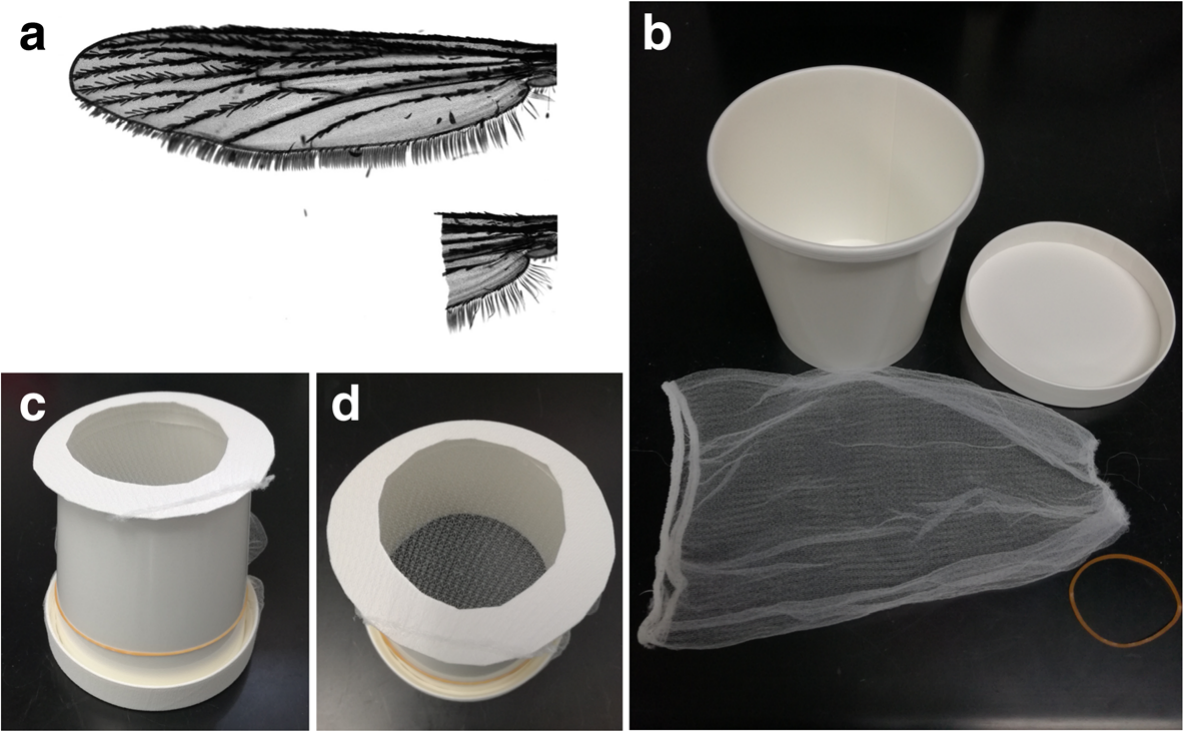
Wing ablation and a rearing container for flightless females. Either wing was cut using micro-scissors leaving the axillary incision (**a**). **b** Materials for a paper rearing cup. Frontal (**c**) and top (**d**) view of the cup

Twenty to 30 mosquitoes 5–7 days after eclosion were collected using an electric aspirator and placed in a paper cup (TOKAN, 31-476-001, top diameter 97.2 mm × height 100.0 mm × bottom diameter 74.3 mm); the top of which was covered with a disposable polyester/polyurethane sink drain net. The mosquitoes were anesthetized in a refrigerator for 5 min and then placed on a cooling plate (Scinics, CP-120) set at 3.5 °C. Using a binocular light microscope, the tip of either wing of a female was held with forceps and the wing was cut at the proximal end (leaving one fourth or one fifth of the wing portion) using micro-scissors (AS ONE, YS-7100BDX) (Fig. 1a). The treatment of mosquitoes on the cooling plate was completed within 6 min, and the wing-cut mosquitoes were immediately transferred to a container customized for the rearing of flightless mosquitoes (Fig. 1b–d and Additional file 1: Figure S1). The mosquitoes for a control group were also anesthetized and kept on the cooling plate for a similar period of time, without any wing ablation.

The customized container was constructed from a cardboard cup (TOKAN, 31-476-001, top diameter 97.2 mm × height 100.0 mm × bottom diameter 74.3 mm), its lid (TOKAN, diameter 97.2 mm), a disposable polyester/polyurethane sink drain net (width 15.5 cm × length 21 cm), and a rubber band. The schematic procedure to fabricate the container is shown in Fig. 1b–d and Additional file 1: Figure S1A. A donut-shaped lid was made by cutting a round hole (approximately 60 mm diameter) in the original lid without the outer frame, and the disposable sink drain net (mesh hole size < 1 mm) was used to cover the hole (Additional file 1: Figure S1B). A cup without a bottom was prepared to insert the net-containing, donut-shaped lid (Additional file 1: Figure S1A and C). The open side of the net was fastened using the outer frame of original lid (Additional file 1: Figure S1D and E). The container was closed with the excess part of the net using a rubber band (Additional file 1: Figure S1F–H).

A cotton pad cut to fit the size of the lid was soaked with 10% sucrose and placed on the container. The cotton pad was avoided from dripping and changed every 3–4 days to fend off fungi contamination. The container was kept inside a plastic box (length 23.8 × width 15 × height 16.4 cm) as a secondary security barrier. The box was kept in an incubator set at 27 °C with humidity over 90% in cycles of 12 h light and 12 h darkness.

### Observation of flight ability and posture

**Fig. 2:**
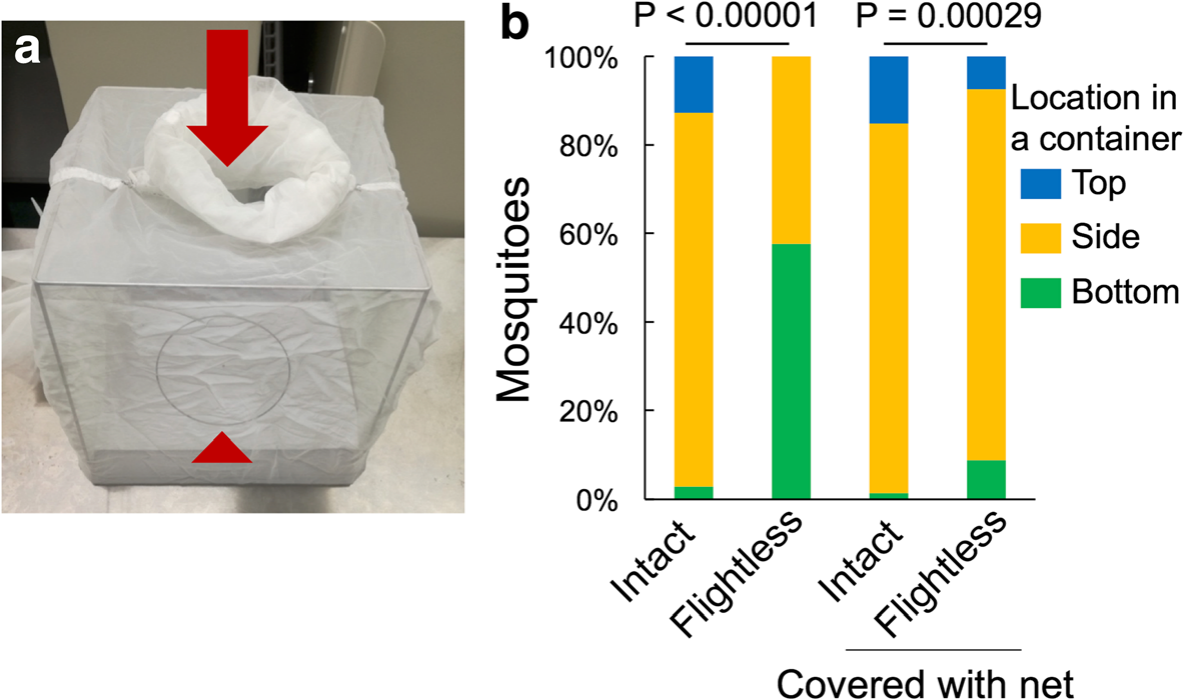
Motility of flightless females. **a** Test cage (bottom 27 × 27 cm, top 25 × 25 cm, height 27 cm) in which females were released individually from the center of the top (arrow) to examine their flight ability. The number of mosquitoes dropped inside the circle (15 cm diameter) at the bottom (arrowhead) was counted (see Table 1). **b** The posture of mosquitoes in a rearing container in which the inside walls were covered with or without a net. Statistical differences were evaluated using the chi-square test

A single mosquito 1 day after wing ablation or a control intact mosquito was collected in an aspirator and released from the top center of a mosquito cage (bottom 27 cm × 27 cm, top 25 cm × 25 cm, height 27 cm). The number of mosquitoes dropped inside a 15-cm-diameter circle drawn on paper, which was laid in the center of the bottom of the cage, was counted (Fig. 2a).

To observe the body posture of intact or flightless females in a resting state, about 20–30 mosquitoes were collected in a customized container covered in all inside surfaces with a net. The container was tapped a few times to make all the mosquitoes release hold of the wall. Five minutes later, the mosquitoes resting at the wall (top, side, or bottom) were observed for 10 s.

### Blood feeding

The mosquitoes 1 day after wing surgery and the controls were fed on blood from mice through an artificial feeding system. Briefly, the blood of male BALB/cAJc1 mice was freshly prepared on the day of feeding by using a syringe loaded with an aliquot of 1 mg/mL heparin in PBS. The equipment for blood feeding was a 25 cm^2^ cell culture flask (Thermo Fisher, 156367) of which one side was covered with Parafilm M (Bemis, PM996) stretched to about four times its original area. Mice blood (2 mL) was applied into a space between the Parafilm M and the surface of the flask using a syringe. The flask itself was filled with a warmed (42 °C) solution of 100% glycerol (Wako), which was changed every 30 min to maintain the temperature of the blood. A container with ~ 30 mosquitoes was placed in a transparent bucket (top diameter 31 cm × height 27 cm × bottom diameter 22 cm) into which CO_2_ was supplied through a CO_2_ pad fixed under the lid to activate the blood foraging of mosquitoes. The blood feeder was placed onto the mosquito container for 1 h, ensuring that the membrane side was in direct contact with the net.

### Measurement of the amount of blood ingested by mosquitoes

Immediately after blood feeding, all the mosquitoes were anesthetized using CO_2_ and the number of the fully engorged mosquitoes was counted. Each blood-fed mosquito was then individually placed in a weighed PCR tube and compared with control unfed mosquitoes. The weight of each tube containing a single mosquito was measured using a microbalance (Sartorius, MSA6-6S-OTR-DM). The amount of ingested blood was estimated by subtracting the mean weight of the control unfed mosquitoes from the weight of engorged ones.

### Egg collection

One day after blood feeding, only fully engorged females were collected and placed into a mosquito container. Two days after blood feeding, each mosquito was individually transferred into a polystyrene vial (Chiyoda Science, KBF-1S, diameter: 25 mm × height: 96 mm) in which a cellulose acetate plug (Fisher Scientific, AS271) filled with RO water was squeezed into the bottom and a piece of filter paper (Whatman, grade 1) was then placed on the plug. The vial was closed with a cotton pad soaked with 10% sucrose. Two days after transferring, the number of eggs laid on the filter paper was counted.

### Longevity test

Twenty to 30 fully engorged females were transferred into a mosquito container, which was placed in a plastic box. To avoid contamination by fungi and other microbes, a sheet of wrap was seated on the bottom of the box and changed occasionally to keep the inside of the box clean. Sucrose solution (10%) was supplied with a cotton pad placed on the top of the container and changed every 3–4 days. Another cotton pad, which was only filled with RO water, was set on the bottom of the container for mosquitoes to lay egg on. Six to 8 days after blood feeding, the cotton pad on the bottom was replaced with new one with 10% sucrose solution to increase the opportunity for nutrient uptake by aged mosquitoes. The sucrose solution was changed every 3–4 days. The box with a mosquito container was kept in an incubator set at 27 °C with humidity over 90% in cycles of 12 h light and 12 h darkness.

### Statistical analysis

All statistical analyses (chi-square test, Student’s *t* test, Kaplan–Meier log-rank test) were performed using R version 3.4.1 (www.r-project.org).

## Results

The flight ability of *A. aegypti* mosquitoes after wing removal was estimated by releasing mosquitoes from the top of a cage and observing their landing sites (Fig. 2a and Table 1). The intact females changed their flying direction immediately after being released from an aspirator and flew to the side or top of the cage. None of the intact mosquitoes landed on the bottom floor of the cage. The wing-ablated mosquitoes dropped directly from the releasing point (Table 1). These mosquitoes failed to even briefly float up in the air, indicating that the flight ability of these wingless mosquitoes was completely abolished. In a conventional paper cup for rearing mosquitoes, most of the flightless mosquitoes walked around at the bottom of the container or stayed on the lower area on the side wall, because it was not feasible for the mosquitoes to climb up (Fig. 2b). No mosquitoes reached to the top of the container, demonstrating the low motility of the flightless mosquitoes (Fig. 2b). To minimize possible physiological stress due to alteration of the mosquito’s posture (from vertical to horizontal) during resting, one wall of the container was then covered with a net stretched to approximately 1-mm mesh, which could help the mosquitoes cling to the wall (Fig. 1c, d and Additional file 1: Figure S1). This modification eventually mitigated the difference in posture between intact and flightless mosquitoes (Fig. 2b).

**Table 1:** Flight ability test of mosquitoes after wing ablation

Wing	Dropped inside circle	Total
Intact	0	103
Cut	101	101

The blood feeding capacity of the flightless mosquitoes was examined by supplying blood from mice using a parafilm-based, artificial feeding system. The blood feeder was placed either at the top or bottom of the mosquito container, and the blood sucking behavior of female mosquitoes was stimulated using CO_2_ supplied from the top. The feeding efficiency of the flightless mosquitoes from the top of the container, where they climbed the netted wall well and reached the blood feeder, was similar to that of the control mosquitoes, whereas feeding from the bottom was insufficient for flightless mosquitoes (Table 2). A previous report showed that *A. aegypti* females in right-side-up or nose-up positions fed less on blood meals than mosquitoes in an upside-down position [4], and the present results were in agreement on the optimum way to serve blood, even to wing-ablated mosquitoes.

**Table 2:** Blood feeding efficiency of flightless females

Females	Blood feeding from	Blood-fed	Total	Ratio
Intact	Top	157	204	0.77
Intact	Bottom	152	205	0.74
Flightless	Top	145	204	0.71
Flightless	Bottom	80	203	0.39*

The significance of differences was evaluated using the chi-square test (*P < 0.01)

**Fig. 3:**
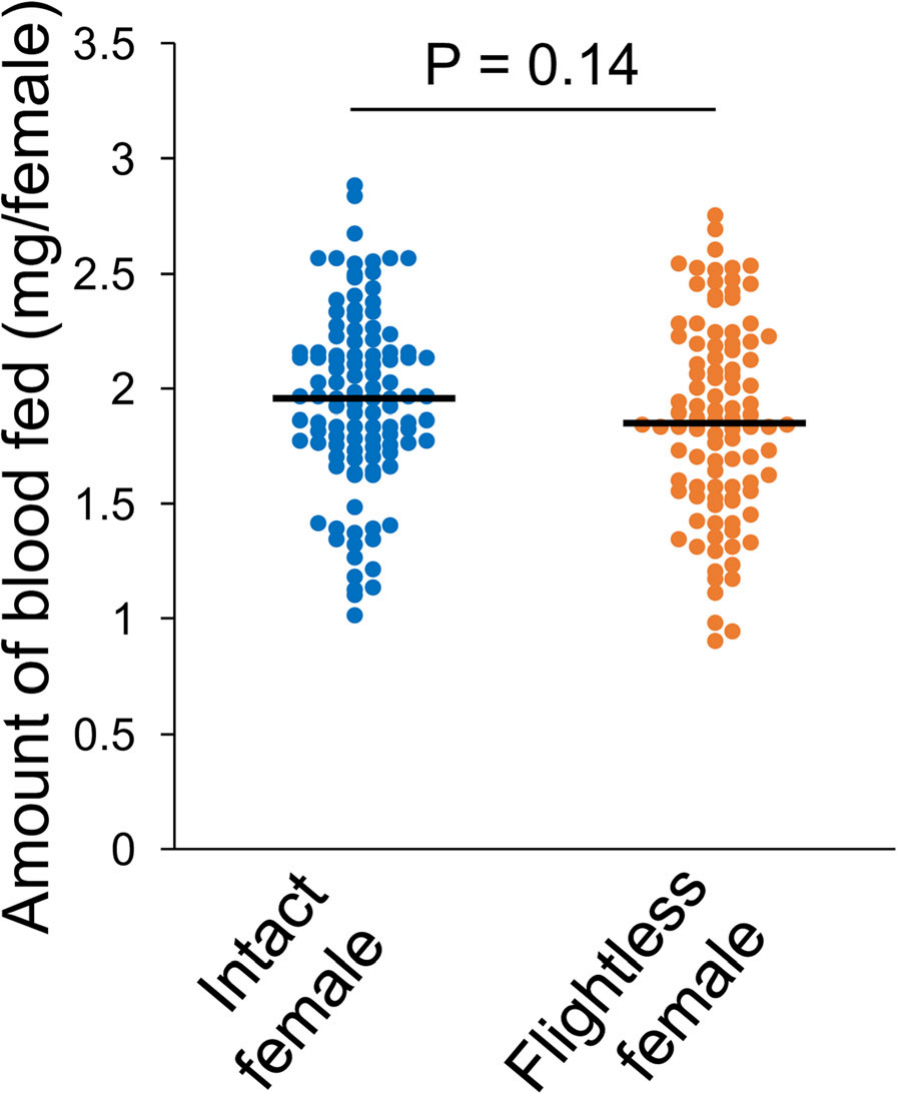
Amount of blood consumed by flightless females. Amount of blood consumed by intact or flightless females using the artificial blood feeding system for 1 h. *n* = 110 for intact females, and *n* = 100 for flightless females. The significance of differences was calculated using Student’s *t* test

**Fig. 4:**
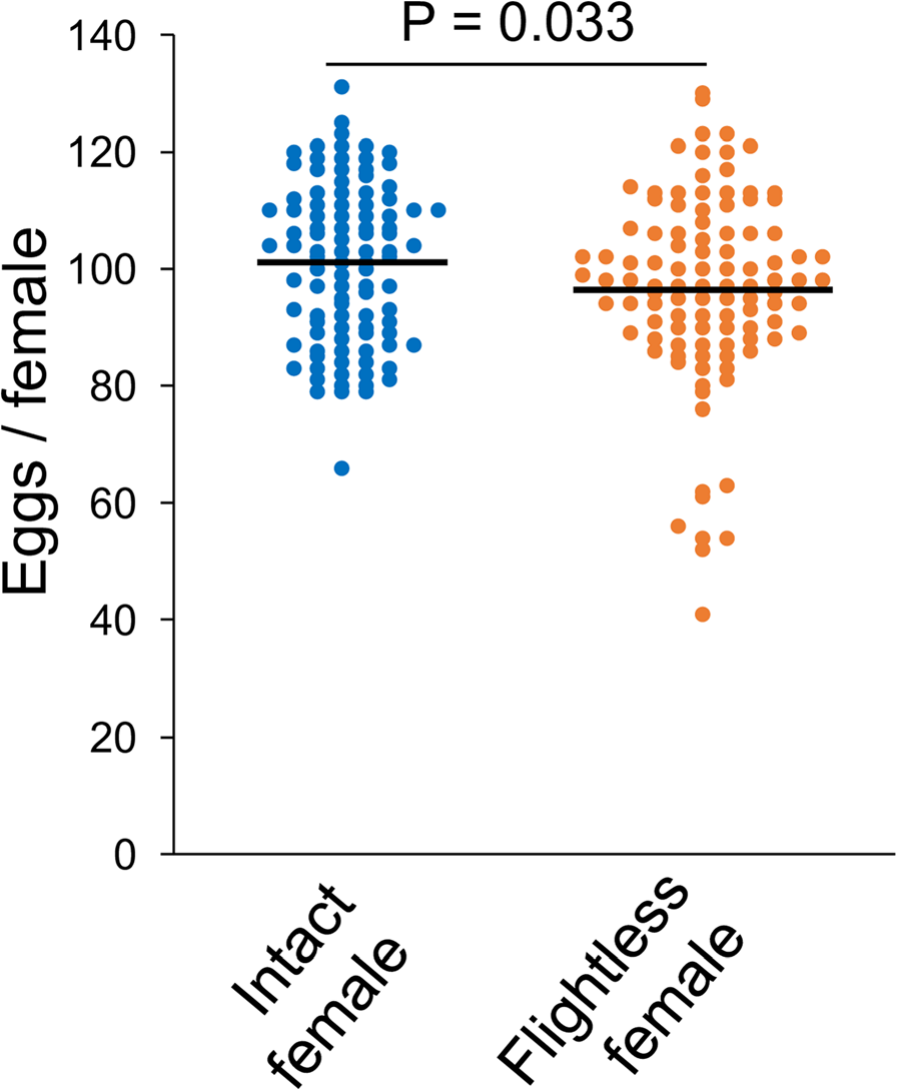
Number of eggs laid by flightless females. The number of eggs laid by intact or flightless females was counted individually. *n* = 101 for intact females, and *n* = 105 for flightless females. The significance of differences was calculated using Student’s *t* test

The amount of blood ingested by the flightless females was similar to the intact ones (mean ± SEM, intact female 1.95 mg ± 0.04, flightless female 1.86 mg ± 0.04, *P* = 0.14) (Fig. 3), suggesting that any physiological damage and/or alterations resulting from ablation of either wing did not have a significant effect on the achievement of blood feeding. The number of eggs laid by the flightless females was slightly but significantly reduced (mean ± SEM, intact female 101 eggs ± 1, flightless female 96 eggs ± 2, *P* < 0.05) (Fig. 4). In the previous study, the amount of blood ingested correlated with the number of eggs laid by individual female mosquitoes [5]. These results suggested that flightless females with similar blood sucking activity may have a defect in either egg production or egg laying behavior.

**Fig. 5:**
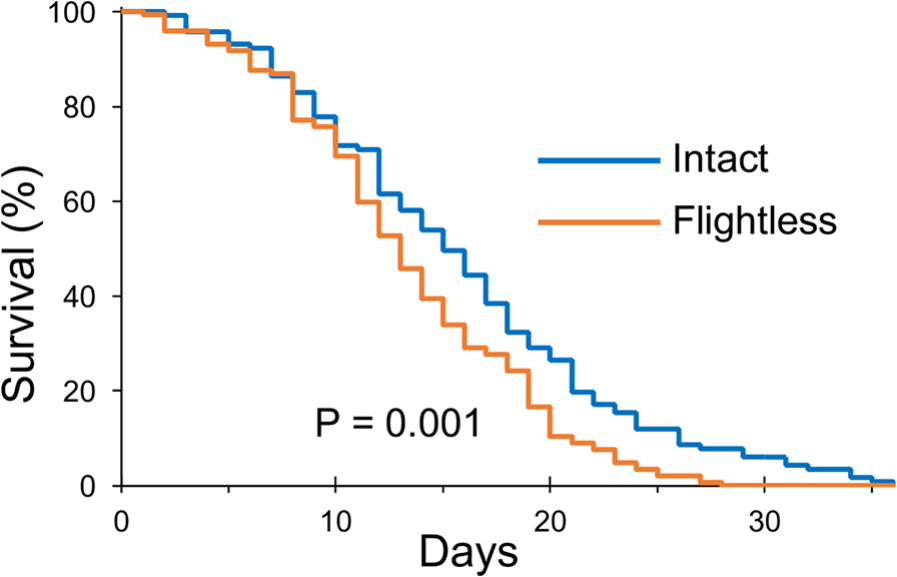
Survival of flightless females after blood feeding. Survival fractions of intact or flightless females fully engorged with blood from mice. *n* = 117 for intact females (blue line), and *n* = 144 for flightless females (orange line). The significance of differences was analyzed using the Kaplan–Meier log-rank statistical test

The flightless females survived for a shorter period than the intact females, which was apparent around 10 days after the start of incubation. Kaplan–Meier survival curves for intact and flightless female mosquitoes showed a significantly (log-rank *χ*^2^ = 10.7, *P* < 0.01) shorter life span for flightless mosquito (Fig. 5). The longevity of *Aedes* mosquitoes is affected by several extrinsic and intrinsic factors [6–8]. The flightless females were located at the bottom of the container more frequently (Fig. 2b), forcibly retaining their altered posture. Incubation of the flightless females in a container without a net covering the side wall forced these mosquitoes to stay around the bottom, which caused strikingly higher mortality (intact 0.88, flightless 0.35, *P* < 0.01) (Table 3). These data suggested that rather than damage from the injury of wing ablation, accompanying behavioral restrictions could be the major cause of the increased mortality rate.

**Table 3:** Survival of flightless females in 8 days after blood feeding

Females	Net covering wall of a container	Alive	Total	Survival
Intact	Yes	84	106	0.79
Intact	No	91	104	0.88
Flightless	Yes	91	107	0.85
Flightless	No	38	109	0.35*

The significance of differences was evaluated using the chi-square test (*P < 0.01)

## Discussion

The secure method for laboratory experiments using pathogen-infected mosquitoes shown in this report provided a reliable and cost-effective way to securely contain pathogen-infected females. Analyzing emerging and re-emerging arboviruses, such as Zika and dengue virus, in *Aedes* mosquitoes is indispensable for comprehensive understanding of pathogen–vector interactions, which could contribute to establishing effective management strategies of infectious diseases caused by arboviruses. The artificial blood feeding method is a versatile system for introducing a variety of arboviruses into vector mosquitoes. The advantages derived from the blood sucking method can enhance knowledge about arbovirus dynamics in mosquitoes. Histological and cytopathological analyses have shown the specific temporal and spatial distributions of arthropod-borne viruses in mosquitoes [9–15] and detailed evolutional analyses of viral genomic RNAs during the course of traversal across mosquito tissues revealed selective pressures from imposing repeated population bottlenecks [16, 17]. To increase the safety of mosquito handling during and after artificial blood feeding, removing mosquito wings is not a completely novel idea because it is quite obvious that wingless mosquitoes are unable to fly anymore. The fragility of mosquitoes, however, has often been problematic when applying physical manipulations such as wing ablation to mosquitoes [18, 19]. To maintain partially disabled mosquitoes alive for long enough for various experiments, a mesh net was employed to cover one wall of the mosquito reservoir intramurally, providing adequate scaffolding for mosquitoes to hold on to. Although advanced analyses related to mosquito flying, e.g., analysis of courtship behavior, cannot be accomplished, the flightless females are extremely safer even outside the special mosquito reservoir when handled with lab coat and gloves following a standard procedure and a diverse range of pathogen–vector interactions can be explored using this method. Practically, the slightly higher mortality of flightless females observed in our study would not compromise the observation of virus behavior in mosquitoes, including virus proliferation and dissemination, because it takes up to 2 weeks for mosquitoes to become competent to transmit viruses after ingestion of a viremic blood meal [20–22]. Although the egg hatching rate is remained to be examined, the fertility of flightless females was sufficient to lay eggs and should contribute to elucidating the interactions of pathogens with the host reproductive system, e.g., vertical transmissions of flaviviruses [23–25]. The applicability of this method should not be limited to cover only *Aedes* mosquitoes, rather other mosquito species transmitting pathogens, although modifications of this method might be required to adapt to species-dependent characteristics [4].

## Conclusions

Although further examination regarding the pathogen infection in the flightless mosquitoes remains to be elucidated, the mosquito rearing method for *Aedes* species presented here was extremely secure, and the protocol was inexpensive and less labor intensive, making it possible to perform intensive studies using mosquitoes carrying human hazardous pathogens, even in counties where highly secure containment systems are mandatory. This protocol could also be applicable to *Anopheles* and *Culex* mosquitoes, which are major vectors for other devastating diseases, such as malaria, West Nile fever, and Japanese encephalitis.

## Additional file

Additional file 1: **Figure S1.** A detailed protocol for construction of a mosquito-rearing container. (A) The bottom of a cardboard cup was cut off. (B) A round plate was separated from the rim of the lid, and the center of the round board was cut out. The donut-shaped lid was placed into a disposable polyester/polyurethane sink drain net. (C) The lid was set on the cup ensuing all the inside area was covered with the net. (D) The net was turned over and fixed using the rim (E). The container was enclosed by the remaining part of the net using a rubber band (F–H). (TIF 1692 kb)
