# Supplementary figures and images for: A highly secure method for rearing Aedes aegypti mosquitoes
Source: Trop Med Health. 2018 May 23;46:16. doi: 10.1186/s41182-018-0098-5 (PMC5966851; doi:10.1186/s41182-018-0098-5)

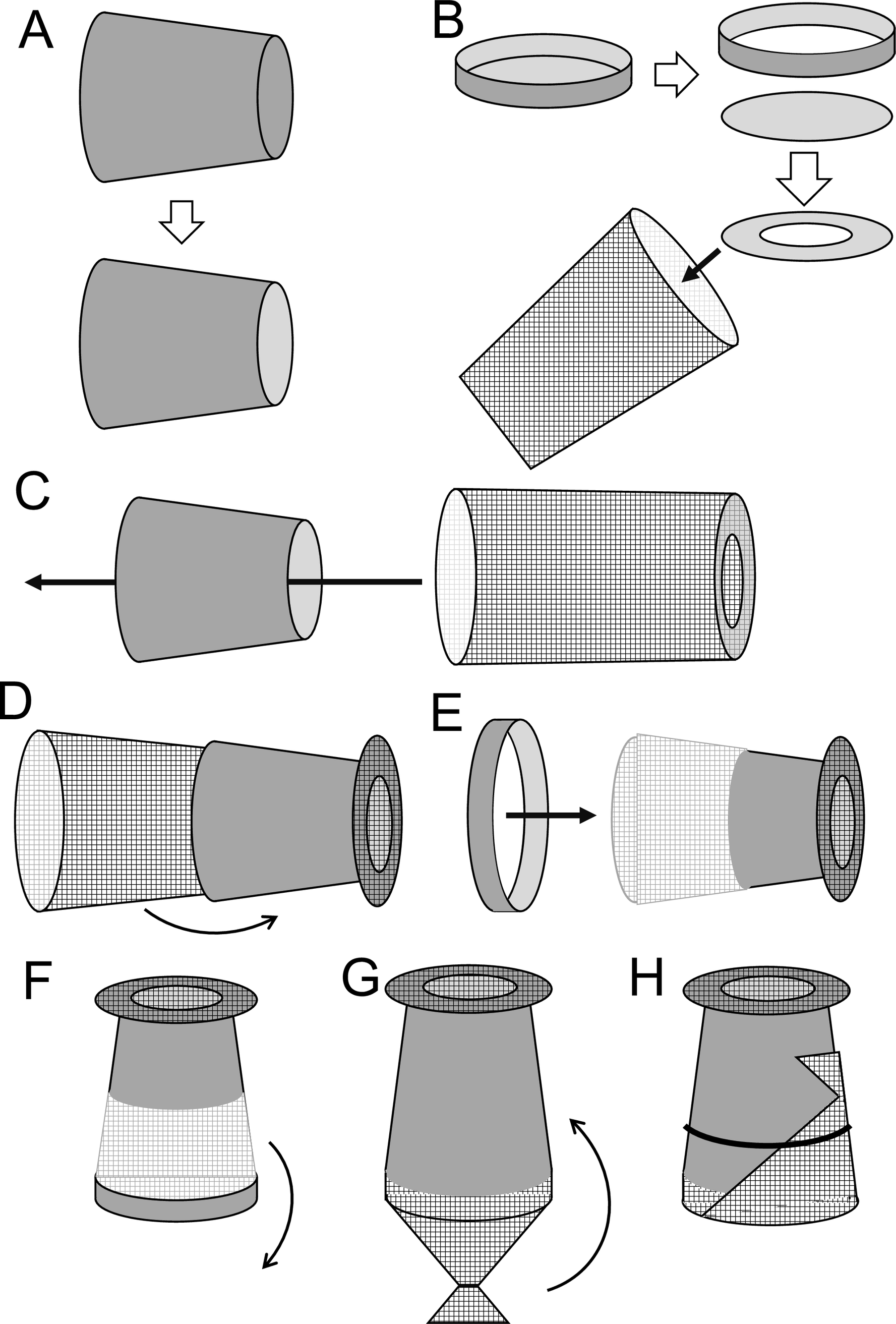

Supplement: Supplementary file 1 — Figure S1. A detailed protocol for construction of a mosquito-rearing container. (A) The bottom of a cardboard cup was cut off. (B) A round plate was separated from the rim of the lid, and the center of the round board was cut out. The donut-shaped lid was placed into a disposable polyester/polyurethane sink drain net. (C) The lid was set on the cup ensuing all the inside area was covered with the net. (D) The net was turned over and fixed using the rim (E). The container was enclosed by the remaining part of the net using a rubber band (F–H). (TIF 1692 kb) [file 41182_2018_98_MOESM1_ESM.tif]
